# CD4+ T Cells: Multitasking Cells in the Duty of Cancer Immunotherapy

**DOI:** 10.3390/cancers13040596

**Published:** 2021-02-03

**Authors:** Jennifer R. Richardson, Anna Schöllhorn, Cécile Gouttefangeas, Juliane Schuhmacher

**Affiliations:** 1 Department of Immunology, Institute for Cell Biology, University of Tübingen, 72076 Tübingen, Germany; jenny@richardsons.de (J.R.R.); anna.schoellhorn@uni-tuebingen.de (A.S.); juliane.schuhmacher@uni-tuebingen.de (J.S.); 2 Cluster of Excellence iFIT (EXC2180) “Image-Guided and Functionally Instructed Tumor Therapies”, University of Tübingen, 72076 Tübingen, Germany; 3 German Cancer Consortium (DKTK) and German Cancer Research Center (DKFZ) Partner Site Tübingen, 72076 Tübingen, Germany

**Keywords:** CD4+ T cells, tumor, immunotherapy

## Abstract

**Simple Summary:**

T cells bearing the co-receptor CD4 on their cell surface are a heterogeneous group of T lymphocytes that exert pro- or anti-inflammatory functions. Evidence from mouse models and cancer patients reveal that various CD4+ T cell subsets play an antagonistic role in the antitumor immune response. This review summarizes current knowledge on CD4+ T cell subsets, on how they impact tumor growth in patients, and which role these cells play in newest cancer immunotherapies.

**Abstract:**

Cancer immunotherapy activates the immune system to specifically target malignant cells. Research has often focused on CD8+ cytotoxic T cells, as those have the capacity to eliminate tumor cells after specific recognition upon TCR-MHC class I interaction. However, CD4+ T cells have gained attention in the field, as they are not only essential to promote help to CD8+ T cells, but are also able to kill tumor cells directly (via MHC-class II dependent recognition) or indirectly (e.g., via the activation of other immune cells like macrophages). Therefore, immunotherapy approaches have shifted from only stimulating CD8+ T cells to targeting and assessing both, CD4+ and CD8+ T cell subsets. Here, we discuss the various subsets of CD4+ T cells, their plasticity and functionality, their relevance in the antitumor immune response in patients affected by cancer, and their ever-growing role in therapeutic approaches for human cancer.

## 1. Introduction

In the last decade, T cell-based immunotherapy has evolved into an established therapeutic tool in oncology. Alongside this milestone, efforts in translational research have been boosted for further improving cancer therapies, extending the cancer types that can be successfully treated, and providing individualized treatments tailored to patients’ needs. Main strategies currently followed are the use of checkpoint blocking antibodies, adoptive transfer of T cells, and cancer vaccines.

Initially, CD8+ cytotoxic T cells were the focus of all these strategies, as these cells have the capacity to directly kill tumor cells. However, CD4+ T cells should not be underestimated. The development of antitumor CD8+ T cells depends on CD4+ T cell help, which involves both dendritic cell (DC) licensing [1,2] and the production of interleukin (IL)-2, the main growth factor for T cells. More recently, it has become clear that CD4+ T cells also have the ability to kill tumor cells directly and to coordinate the antitumor function of innate immune cells such as macrophages [3,4]. Research on the impact of CD4+ T cells in the antitumor response is complicated by the fact that a variety of CD4+ T cell subsets with different functions have been described. In addition, differentiated CD4+ T cells are very much sensitive to extrinsic signals and are able to quickly shift their effector state, a phenomenon that is referred to as CD4+ T cell plasticity (reviewed in [5,6,7]). Finally, identification and characterization of antigen-specific CD4+ T cells is more difficult than that of CD8+ T cells, due to the absence of strict major histocompatibility complex (MHC)-class II binding motifs and the promiscuity of peptide binding on several MHC-class II allelic products. All this needs to be considered when studying the interplay of CD4+ T cells and tumors, and when thinking of how to exploit CD4+ T cells in immunotherapy approaches.

## 2. Basics on CD4+ T Cells: Commitment, Plasticity, and Functions

### 2.1. Priming of CD4+ T Cells 

Upon activation by professional antigen-presenting cells (APCs), like DCs, naïve CD4+ T cells differentiate into various T effector cell subsets, and into memory CD4+ T cells. This polarization is crucial for the immune response and is dependent on three complementary signals: the interaction of the T cell receptor (TCR) of the naïve CD4+ T cell with the peptide:MHC-class II complex on the APC; a costimulatory signal, typically transduced through binding of CD28 on the T cell with its ligands CD80/CD86 on the APC; and the composition of the cytokine milieu. Cytokines, mainly produced by APCs, are well known for their dominant role in CD4+ T cell fate. TCR signaling strength, and the T cell costimulatory molecules and their interaction with specific ligands also influence lineage differentiation. As an example, a strong TCR-mediated signal promotes predominantly T helper (Th) 1 differentiation, while the Th2, follicular helper (Tfh), or memory T cell differentiations are favored upon weak signaling [8,9]. The TCR signaling strength is regulated by the affinity of the peptide: MHC-class II and/or by the quantity of peptide:MHC-class II complexes (peptide load) on the APC surface [8]. Moreover, the costimulatory signal may influence polarization depending on the nature of the costimulatory molecule. As an example, OX40 triggering drives Th2 differentiation [10,11]. The highly complex process of CD4+ T cell polarization is therefore driven by multiple signals, whereby the individual signals are probably hierarchically and temporarily apart to give rise to the various CD4+ T effector cell subsets. Hence, the sequential order of these signals might impact the efficacy of the T cell priming. For example, pre-exposure to a high amount of IL-2 was shown to prevent TCR-dependent activation of CD4+ helper T cells [12].

### 2.2. Effector CD4+ T Cell Subsets and Their Identification

CD4+ T cells come in “many flavors”. The main CD4+ T cell subsets are Th1, Th2, Th17, Th9, Th22, induced or natural regulatory T cells (iTregs and nTregs), and Tfh cells; their characteristics are summarized in Table 1 [13,14,15,16,17,18,19,20]. Many of these subsets can be derived through in vitro cultures of uncommitted precursor cells in the presence of cocktails of cytokines. Importantly, some cytokines secreted by one lineage act to inhibit alternate differentiation. Altogether, three dominant cytokines IL-12, IL-23, and transforming growth factor-β (TGF-β) have been proposed to control CD4+ T cell fate [14].

CD4+ lineages are defined by the expression of lineage-defining transcription factors (TFs, or master regulators) that promote their characteristic phenotype. Specific signal transducer and activator of transcription (STAT) proteins, a pattern of chemokine receptors reflecting different homing properties, and various cytokine production profiles contribute to their different functions [13,14]. Intracellular flow cytometry staining is one of the most popular methods for addressing multi-cytokine production and simultaneously identifying the producer cell subsets. Cell surface markers can also be used to substantiate subset characterization, and are also included in Table 1.

In the context of antitumor T cell immunity, mainly Th1, Th2, Th17, and Tregs have been characterized and discussed. This, however, does not exclude that other subsets may be involved either in promoting, or in contrast, inhibiting innate or adaptive immune effectors of the antitumor response. Some examples will be discussed in the next section.

Differentiation into Th1 cells is induced by IL-12 and interferon-γ (IFN-γ). Th1 cells express the TF T-BET, which drives IFN-γ production. Together with IL-2 and tumor necrosis factor (TNF), IFN-γ activates macrophages and the recruitment, expansion, and functions of cytotoxic CD8+ T cells (CTLs). Th1 cells are well known to control immune effector functions against intracellular pathogens [13,14].

Th2 cell differentiation is mediated by IL-4 as the major cytokine driving GATA3 expression, the lineage-specific Th2 TF. IL-4 is not commonly produced by DCs, but by some innate lymphocytes and basophils. In addition, alarmins produced by epithelial cells, like IL-33 or IL-25 have been proposed to directly (Th2 cells can express cognate receptors) or indirectly (via the activation of other immune cell types like DCs or innate lymphocytes) promote Th2 differentiation. Th2 cells mediate effector functions against extracellular parasites and activate tissue repair via the secretion of the anti-inflammatory cytokines IL-4, IL-5, IL-9, and IL-13 (for a recent review on Th2 cells, see [11]).

Priming in the presence of TGF-β and IL-6, but also IL-1β, IL-21, and IL-23 drives differentiation towards Th17 cells, leading to the expression of the TF retinoic acid receptor-related orphan receptor (ROR)-γt. Th17 cells are essential for immunity against extracellular pathogens, like bacteria and fungi, and mediate their proinflammatory function by secretion of IL-17A and F, IL-21, IL-22, and CC-chemokine ligand (CCL) 20 [21].

TGF-β in combination with IL-2, and in the absence of IL-6, drives the differentiation of iTregs expressing FOXP3. iTregs are phenotypically CD4+CD25+CD127 (the α-chain of the IL-7 receptor) low/−, and generally FOXP3+ [22]. Tregs can mediate their suppressive function via a range of mechanisms, including the secretion of immunosuppressive factors like IL-10 and TGF-β or the local deprivation of factors essential to effector T cells, such as IL-2 or ATP. Additionally, Tregs express inhibitory checkpoint molecules like CTLA-4 or TIGIT that can contribute to contact-dependent inhibition of immune effector cells [21]. 

### 2.3. CD4+ T Cell Memory and Cytotoxic Potential

Once the effector phase of a T cell response terminates, a small fraction of long-lived memory T cells remains. In the blood, memory T cells can be classified into central memory (T_CM_), effector memory (T_EM_), and effector memory cells re-expressing CD45RA (T_EMRA_), which all are thought to be derived from stem cell-like precursors (T_SCM_). Within tissues, resident memory cells (T_RM_) have also been described [13,19]. The main phenotypic markers for each of these differentiation stages are listed in Table 1.

Naïve T cells (T_N_) can be identified by the expression of CD45RA together with the chemokine receptor CCR7, CD62L, and CD27, and the lack of CD45RO, whereas memory T cells usually lack CD45RA [23]. Originally, two CD4+ memory subsets have been discriminated in the blood [24]: CD45RA-CCR7+ T_CM_ have the capacity to home in to lymph nodes, a high potential for secondary expansion, but weak immediate functionality; CD45RA-CCR7− T_EM_ exert immediate effector functions and produce various cytokines e.g., IL-4, IL-5, and IFN-γ. Although rare in the periphery of most healthy individuals, CD4+ T_EMRA_ cells have been identified in the context of viral infections where they are associated with virus control [25,26].

In addition to T_EM_ cells found in the blood, a subset of tissue-resident CD4+ T_EM_ cells has been described within several organs such as the skin, the gut, or the lung. Like CD8+ T_RM_, which have been characterized in more detail so far, these cells express specific surface molecules like the integrin CD103 and/or the activation marker CD69, and are thought to serve as a first-line defense in barrier organs (for a recent review on CD4+ T_RM_ cells, see [27]). Whether they can be targeted in cancer immunotherapy is being discussed [28]. Another memory subset that could be favorably exploited in oncology is T_SCM_, since these cells are long lived, capable of self-renewal, and able to give rise to T_CM_, T_EM_, and effector T cells. T_SCM_ mainly display a naïve-like phenotype (CD45RA+CCR7+CD62L+CD27+, and CD28+), but express markers of activated T cells like CD95, CXCR3, or elevated levels of LFA-1 [29].

Although they are less recognized than their CD8+ counterparts so far, CD4+ CTLs appear to be bona fide killer cells able to secrete granzyme B and perforin and also capable of killing target cells in a MHC-class II restricted manner [30,31]. After activation and degranulation, they express LAMP-1 (CD107a) at the cell membrane, as their CD8+ CTL counterparts do [20,26]. MHC class I-restricted T cell–associated molecule (CRTAM) has also been proposed as a marker for identifying CD4+ CTLs [32]. CD4+ CTLs appear to be highly or terminally differentiated effector cells (at least of the Th1 subset) and are found mostly in T_EMRA_ subpopulations [20,33]. These cells have been essentially described in the context of virus infections [26,30,31].

### 2.4. Plasticity of CD4+ T Cells 

CD4+ T cell differentiation into the various subsets was originally believed to be a terminal event. This scholastic model of an irreversible T cell lineage differentiation has been considerably revised in recent years. Numerous studies have shown that T cell subsets are not at all terminally differentiated but rather plastic and able to acquire different properties and functions particularly in the setting of secondary immune responses (reviewed in [28]). Loss of FOXP3 expression by Tregs has been described as well [5,34], and especially FOXP3+ Tregs and Th17 helper cells show high plasticity and are able of reciprocal differentiation. Further, Th17 cells can also gain a Th1 phenotype [7]. Beyond that, cells of various Th subsets were shown to not only express their lineage-specific cytokines and TFs, but simultaneously those of other Th lineages, making them similar to hybrid cells [5]. Under certain circumstances, even helper cells can acquire suppressive hallmarks, like Th1 cells that produce IL-10, a possible self-regulation mechanism to prevent immunopathology [35]. IL-12 can induce IL-10 in Th1 clones, which was shown to regulate overshooting immune responses, indicating that IFN-γ/IL-10 producing Th1 cells have a regulatory function. Those cells were named type 1 regulatory (Tr1)-like T cells and are crucial in maintaining tissue integrity.

Plasticity and flexibility of CD4+ T cells are influenced by a number of extrinsic (e.g., cytokines) and intrinsic factors that are all far from being identified. STAT proteins control the expression of lineage-specific TFs, and also drive epigenetic changes like histone modifications or DNA methylations that open certain DNA regions for transcription [36]. In addition, micro (mi) RNAs are involved in the control of CD4+ T cell differentiation. Investigations on the factors involved in Th17 instability are most advanced [37]. Hence, DNA remodeling enzymes and miRNAs might be targets for therapeutic manipulation, but first we need to gain a clearer picture on their target genes and develop highly specific drugs.

Thus, recent studies have shown that Th subsets are more flexible than originally thought. Even if most of these observations derive from in vitro models, they indicate that antigen-experienced human CD4+ T cell subsets are not stiff but plastic and could be manipulated. This is obviously of high importance for translational immunology: on the one hand, it makes our comprehension of antitumor T cell responses more nuanced, from a black-and-white to a more colorful picture; on the other hand, it opens perspectives for manipulating T cell responses ex vivo or in vivo [37]. The obvious application in tumor immunotherapy would be to shift the balance to more Th1, and less Treg cells. Early clinical testing of various classes of TGF-β inhibitors is for example ongoing. Ex vivo engineering of T cells for increasing functionality and lineage stability is also a feasible option to be explored for adoptive cell transfer. 

### 2.5. Lessons from Immunity and Vaccination against Pathogens

Not only the quantity, but also the quality of T effector cells is essential to an effective immune response. Polyfunctional Th1 cells (IFN-γ, TNF, and IL-2 producers) appear to better control virus load (e.g., CMV, HIV, HCV, and Flu) than monofunctional cells, and to contribute to vaccine efficacy [38,39,40,41]. They express higher levels of cytokines per cell, show increased CD40L expression, and most interestingly, are enriched in cytotoxic effectors [42,43]. In yellow fever vaccination, which confers protection for decades, CD4+ T cells respond faster to the vaccine than CD8+ T cells [44], and this could be an important aspect in the development of high-quality immune responses and protection. Moreover, polyfunctional effectors appear to have a distinct transcriptional signature associated with immune activation, cytokine signaling, and lymphocyte chemotaxis, potentially controlled by IL-27 [45]. All these findings suggest that cancer immunotherapy approaches should also strive for stimulating multifunctional effector T cells.

## 3. CD4+ T Cells in Cancer: Multipotent Effectors Orchestrating Antitumor Immunity

The helper functions of CD4+ T cells towards CD8+ T cells are well known: CD4+ T cells are essential for the activation and expansion of CD8+ effectors and for the generation and maintenance of CD8+ T cell memory [46,47]. In the tumor context, they might also participate to epitope spreading and amplification of the CD8+ T cell response.

**Figure 1:**
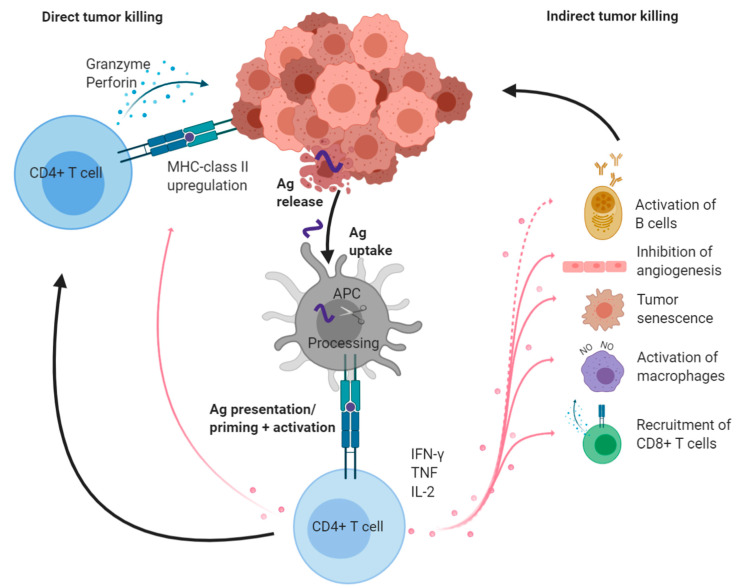
Overview of CD4+ T helper cell tasks in antitumor immunity. After priming and differentiation, effector cytotoxic CD4+ T cells can kill MHC-class II+ tumors directly by, e.g., the release of perforin and granzyme (left). CD4+ Th cells produce IFN-γ, TNF, and IL-2, which have pleiotropic effects on immune or non-immune cells in the TME (right). Activated CD8+ CTLs and NO-producing macrophages participate in tumor elimination and amplify local antitumor immunity via the release of further tumor antigens. IFN-γ and TNF drive tumor senescence and inhibition of angiogenesis. Activated B cells are found in human tumors, however, their exact function is still unclear (dotted line). Created with BioRender. Ag: antigen; NO: nitric oxide.

In addition, CD4+ T cells have the ability to control tumor growth in different ways, even in the absence of MHC-class II on the tumor cells [48,49] (Figure 1). MHC-class II molecules are preferentially expressed on APCs and neoplasms derived from APCs. However, tumors from other origins can express relevant amounts of MHC-class II molecules, most likely in relation to inflammatory conditions within the tumor microenvironment (TME). Such MHC-class II+ tumors can be directly killed by CD4+ CTLs [3,50]. For the elimination of MHC-class II negative tumor cells, CD4+ T cells display a number of other functions. The most important cytokines involved in this indirect control pathway are TNF and IFN-γ (both mainly produced by Th1 cells), which can act on immune and non-immune target cell types (Figure 1). CD4+ T cell effectors can activate M1 tumor-associated macrophages (TAMs) to produce nitric oxide (NO) [4,51], or partner with natural killer (NK) cells [48]. Tumor development and spreading can be impaired either via inhibition of angiogenesis [52] and/or induction of growth arrest, known as tumor senescence [53]. Whether one of these mechanisms dominates the antitumor CD4+ T cell response in certain tumors at certain stages, or rather several of them can be involved simultaneously in individual tumors is not known.

Tumor antigens that are recognized by CD4+ T cells can be non-mutated or mutated. Some examples in cancer patients will be given below, but according to mouse experiments, neoantigen recognition appears to play a major role in tumor control [54].

Most of the reports describing these modes of action of antitumor CD4+ T cells are based on mouse or in vitro models. However, is there any evidence that CD4+ T cells indeed play a relevant role in cancer patients?

### 3.1. Evidence from Human Tumors

The more informative approach is to “look within tumors”, i.e., to assess immune cell infiltrates in tumor tissues collected from large patient cohorts. This can be done by immunohistochemistry and real-time PCR analysis of surgically removed tissues. In early seminal observations, lymphocytic infiltration into tumor tissues had been related to better prognosis [55,56]. Later, intra- or peri-tumoral CD3+ T cells, CD8+ T cells, Th1 cytokines (especially IFN-γ), and an “inflamed gene signature” were also shown to associate with improved clinical outcome [57,58]. As recently reviewed [59], it is currently recognized that the presence of Th1 (and IFN-γ) within tumor-infiltrating lymphocytes (TILs) generally associates with a better clinical outcome. The picture is not as clear for Th2, Th17, or even Treg cells. Intratumoral expression of GATA-3, the Th2 TF, has been associated with either reduced (e.g., pancreatic carcinoma) or enhanced (e.g., breast cancer) survival [60,61]. As another example, Th17 infiltration, alone or together with Th1 TILs, has been associated with either improved (in ovarian or oropharyngeal cancers [62,63]), or with worse (in colorectal carcinoma [64]) prognosis. Depending on the study, Tregs are either favorable, unfavorable, or do not have any impact on patient survival [59]. Methodological tools (e.g., whether anti-FOXP3 antibodies (Abs) alone or together with CD25 Abs are used for Tregs identification) might explain, at least partially, these apparently contradictory observations. Not only the mere number of cells of each subtype, but also their proportion to the other intra- or peri-tumoral subsets may deliver more informative results: for example, the CD8+/Treg cell ratio, rather than each of the two subsets separately, has been shown to predict survival in cervical cancer [65].

Th9 cells are potent antitumor effector cells when adoptively transferred in mice [66], but their impact on human tumors is not yet established. Other potentially relevant cells are cytotoxic CD4+ T cells. Single cell RNA sequencing (scRNA-seq) revealed that several states of cytotoxic CD4+ T cells are present within human bladder cancer and that a specific gene expression signature associates with response to PD-L1 Ab therapy [50]. More studies on the presence and function of CD4+ CTLs within the TME are needed for identifying the exact impact of this population in human cancer.

Various reports point out that virtually all immune cell types can be found within the TME, but we are far from understanding which functions these various cells may exert and how they interact together with growing tumors. In addition, the organization of the immune cells within the tumoral tissue might be a central element for generating a productive immune response. Tertiary lymphoid structures (TLS) are present in many cancer types and can predict disease outcome [67]. TLS not only contain DCs and Th1 cells but also B-lymphocytes and Tfh cells. Both latter types of cells have recently been reported to predict improved outcome, in particular during checkpoint blockade [68,69,70,71]. How B cells could impact cancer growth is not known (Figure 1), but it is well established that IgG Abs directed at numerous tumor-associated antigens are frequently detected in cancer patients.

### 3.2. Antitumor CD4+ T Cells in the Blood of Patients and Systemic Biomarkers of Therapy Responses

Tumor samples are not always available, especially for repeating immune measurements over time. Even if not representing the main “place of action”, blood samples (e.g., liquid biopsies) are easier to collect and handle. Current hopes are that blood could help to identify immune markers that predict clinical response at baseline or at an early time during treatment [72]. From the technical point of view, standardization of blood immune cell testing is highly feasible, and T lymphocytes or other cell subsets can be robustly assessed by multiparametric flow or mass cytometry analyses [15,73].

Naturally occurring CD4+ T cells directed at tumor-associated or -specific antigens can be detected in the blood of cancer patients, but the impact of these T cell responses on clinical outcome is just starting to be investigated. For example, spontaneous Th1 responses against the telomerase reverse transcriptase (TERT) antigen are observed in approximately one-third of patients with non-small cell lung carcinoma (NSCLC, preferentially at localized stage). These cells are associated with increased overall survival (OS), while antiviral T cells in the same patients have no predictive value [74]. Another example is the CD4+ T cell immunity against human papilloma virus (HPV-16): while strong E2- and E6-specific, Th1/Th2 mixed CD4+ T cell responses are frequently detected in healthy women, they are absent or strongly impaired in patients with cervical cancer [75]. Such kind of observations cannot per se conclude whether these tumor-antigen specific T cells are directly involved in tumor control, or if they rather reflect a “fitter” antitumor immunity, but this question can be addressed in vaccination approaches (see next section). In contrast, circulating anti-Melan A CD4+ T cells producing IL-4 or IL-17 were associated with worse 5-year survival in metastatic melanoma patients, while anti-NY-ESO-1 CD4+ T cells (mainly TNF producers) did not have any impact [76].

Another very active area of study is the search for immune cell subsets, which impact a clinical course and/or response to treatment. Markers of T cell function (including Treg populations) and T cell exhaustion (mainly within the CD8+ subset so far) are especially in focus. For example expression of PD-1, TIM-3, and LAG-3 on TILs was significantly associated with shorter survival in several tumor types, and with insensitivity to PD-1 blockade [77,78]. Although others and we found that the expression of checkpoint molecules on CD4+ (and CD8+) blood T cells hardly reflects that of TILs [79,80], it is hoped that meaningful information can still be obtained by analyzing blood cells. 

Among more than 20 immune parameters assessed in the above-mentioned study addressing anti-TERT CD4+ T cell immunity, anti-TERT Th1 responses inversely correlated with the frequencies of CD4+ cells coexpressing PD-1 and TIM-3, and patients with high levels of anti-TERT CD4+ but low CD4+PD-1+TIM-3+ cells had a significantly better prognosis [74]. In another NSCLC study, higher percentages of peripheral CD4+CD62L^low^ T cells (with a Th1 profile) pretreatment were strikingly associated with response to PD-1 blockade, whereas CD25+FOXP3+CD4+ T cells were increased in non-responders. Taking these two subsets into account, the authors proposed a formula for predicting non-responder patients. Moreover, high levels of CD4+CD62L^low^ were maintained in long-responder patients, whereas acquired resistance to therapy was associated with a decrease of this population [81].

Immune biomarkers could be of great help to identify responding patients or to predict toxicities, not only before, but also during the first cycles of treatment. Most studies aiming at identifying such predictive parameters have been performed so far on patients receiving checkpoint inhibitors (CPIs). Changes in absolute counts or frequencies of certain peripheral immune cells and appearance of distinctive phenotypes could indicate the development of a protective immune response [82]. Specifically for CD4+ T cells, elevated frequencies have been described to associate with longer OS in melanoma patients after Ipilimumab (anti-CTLA-4 Ab) therapy [83]. In another recent study in mice and patients, mass cytometry with more than 40 different Abs and systems-wide analysis revealed that tumor rejection upon immunotherapy is accompanied by a number of systemic quantitative and qualitative changes in various immune cell subsets. Strikingly, CD4+ T cells were the main contributors to effective responses. In melanoma patients who received Ipilimumab (together with GM-CSF), elevated levels of CD4+CD127^low^PD-1-cells were found in the blood of responders, but not non-responder individuals [84].

Other current promising strategies for biomarker identification exploit progresses in high-resolution methods such as scRNA-seq or TCR clonotypic analyses [85,86]. All these high-dimensional methods require specific technical expertise, which is available at expert laboratories only, and at this stage not yet harmonized across centers. In the near future, they should allow a comprehensive view on relevant immune cells and on dynamic changes of these cell subsets in the blood upon immunotherapy. They will also reveal if, and to which extent, immune biomarkers are common between tumor types and across therapies, and hopefully define a set of blood parameters that can be broadly assessed in daily practice.

## 4. Harnessing CD4+ T Cells for Immunotherapy

There are many approaches of immunotherapy: checkpoint blockade is the standard treatment for a growing number of cancers, and research on how to combine and/or manipulate further inhibitory or activating T cell co-receptors is actively ongoing [87]. Although logistically more demanding, adoptive transfer of large numbers of in vitro expanded antitumor T cells, either native or genetically modified, is also an option. At the forefront, chimeric antigen receptor (CAR) T cells are already approved for some hematological malignancies [88]. Finally, antitumor vaccines, which are designed to specifically activate patients´ T cells in vivo by administering synthetic peptides, nucleic acids, antigen-loaded APCs, or viral-based vectors are being actively tested [89].

### 4.1. Checkpoint Blockade

One of the main escape mechanisms of tumor cells is the expression of ligands of T cell inhibitory receptors. CPI Abs, which mainly target the inhibitory molecules CTLA-4 and PD-1/PD-L1 so far, enhance antitumor T cell activity, especially in tumors with a high mutational load like melanoma or NSCLC [90,91]. As CD4+ and CD8+ TILs readily express a number of checkpoint molecules [79,80], both T cell types should be reactivated after CPI.

In vitro blocking experiments can be performed, but in general, effects on either CD4+ or CD8+ TIL function are relatively modest. As an example, we could observe modest CD8+, but not CD4+, T cell reactivation after dual PD-1, and LAG-3 Ab blockade in TILs from some renal cell carcinoma patients. Interestingly, treatment with anti-PD-1 also led to upregulation of LAG-3 (but not TIM-3) on both CD4+ and CD8+ TILs within three days [80].

Whether CD4+ T cells, and which subsets thereof, are targeted in cancer patients treated with CPI can be addressed in biomarker studies either within the tumor itself, or in the blood as discussed in the previous section. It is important to note that CTLA-4 vs. PD-1/PD-L1 blockade appears to engage different effector cell subsets, but that a growing number of studies are revealing the role of CD4+ T cells in responder individuals. By analyzing TIL populations with high-dimensional mass spectrometry, Wei et al. observed that both PD-1 or CTLA-4 blockade in mouse tumor models and human melanoma drove the expansion of “exhausted-like” CD8+PD-1+TIM-3+ T cells. In addition, CTLA-4 blockade specifically induced CD4+ effectors with an ICOS+ Th1-like phenotype [92]. ICOS had been shown to be upregulated on TILs and peripheral CD4+ T cells in patients receiving Ipilimumab [93]. In another report, tumor-infiltrating T cells present before and after anti-PD-1 therapy (Pembrolizumab) in patients with basal or squamous carcinoma were compared by scRNAseq combined with TCR clonotyping analysis. The main finding of this study was that checkpoint blockade induced an influx of new T cell clones into the tumor tissue, which were identified as activated/exhausted tumor-specific CD8+ T cells (*PD1+TIM3+CD39+CD103+*); interestingly, the frequency of CXCR5+ Tfh was also increased after treatment [86]. When looking for intratumoral differences between patients responding or not responding to PD-1 Ab treatment, Ribas et al. also found that the frequencies of CD4+ T cells expressing CD57 (a marker generally associated with terminal differentiation and exhaustion of CD8+ T cells) was increased in non-responder TILs, suggesting that CD4+ T cell dysfunctionality associates with tumor progression [94]. Whether CPI also recruits CD4+ CTLs is not yet known, but the expansion of NY-ESO-1 specific, EOMES+ CD4+ T cells able to lyse autologous tumor cells in a MHC-class II restricted manner was described in melanoma patients after Ipilimumab infusion [95].

Finally, Tregs also express checkpoint receptors, especially high levels of CTLA-4. Whether CTLA-4 Abs can lead to Treg depletion in vivo is still discussed, and assessments of Tregs within tumors following therapy led to opposite results [96,97]. Altogether, effects on Tregs seem to be depending on the ability of anti-CTLA-4 Abs to induce antibody-dependent cell-mediated cytotoxicity (ADCC). This is likely governed by their binding to Fc-receptors (FcRs), hence by the Ab isotype (Ipilimumab is an IgG1 isotype), the polymorphism of FcRs, and possibly the presence of certain FcR+ cell types like CD16+ non-classical monocytes [96,98].

### 4.2. Adoptive Transfer Approaches

In early mouse experiments, the adoptive transfer of in vitro expanded tumor antigen-primed T effector cells showed that CD4+ T cells are needed to augment CD8+ T effector responses. CD4+ T cells, moreover, were superior in infiltrating and in proliferating in tumor tissue, highlighting their importance in the local antitumor immunity [99]. TIL adoptive transfer was, together with IL-2 infusion, one of the pioneer anticancer immunotherapies in patients. In most of the ongoing clinical studies, mixtures of in vitro expanded effector T cells are reinfused to the patients. High numbers of cells, with a high proportion of CD8+ T cells, and an effector phenotype have been associated with objective clinical responses [100]. Approximately 20% of TIL products were shown to contain antitumor CD4+ effectors in melanoma patients, although little attention was given to CD4+ T cells in the past [101]. Still, treatment of single patients with clonal or bulk TIL-derived CD4+ T cells already suggests that infusion of large numbers of CD4+ T cells, might, at least in certain cases, suffice for controlling tumor growth. One study reports on a patient with metastatic melanoma who received autologous NY-ESO-1 directed CD4+ cloned T cells and experienced a durable clinical response [102]. More recently, another patient with metastatic cholangiocarcinoma received a TIL product containing CD4+ T cells specific for a mutation in erbb2 interacting protein (ERBB2IP). After initial tumor control, the patient relapsed, and a second cell transfer with enriched anti-ERBB2IP CD4+ T cells led to durable clinical response [103]. Expansion and persistence of CD4+ T cells directed against another tumor-specific mutation (BRAF) has also been observed in another patient who achieved long-term tumor control after TIL transfer [104].

Although it should not be concluded from these few cases that CD8+ T cells are dispensable, they suggest that for certain patients, for example when no autologous CD8+ T cell product with tumor reactivity can be derived, CD4+ T cell-based therapy could be helpful. One key element for clinical effect might be the capacity of transferred CD4+ T cells to induce epitope spreading, and to recruit and support further immune effectors like antitumor CD8+ CTLs.

With the breakthrough technology of using engineered T cell receptors like CARs, new opportunities of adoptive transfer immunotherapy are now available, which are also being extended to the use of CD4+ T cells. CAR T cells generally contain a mixture of transduced CD8+ and CD4+ T cells. The generation of CAR T cells out of blood products harboring a higher frequency of memory CD8+ T cells (CD8+CD45RO−CD27+) and a higher CD4+/CD8+ ratio was associated with better in vivo expansion and clinical response in multiple myeloma patients [105]. Furthermore, preclinical models described that CD4+ CAR T cells persist and keep their effector potency much longer after tumor challenge; they might even outperform CD8+ CAR T cells, especially at high tumor load [106,107]. CD8+ T cell help, production of inflammatory cytokines and lower susceptibility of CD4+ T cells to exhaustion might explain these observations. Indeed, in vitro, CD4+ CAR T cells were shown to exert cytotoxic activity against tumor cells, although at a lower level than CD8+ CAR T cells, but to produce more IFN-γ and TNF, and to proliferate more extensively upon contact with tumor cells [108].

Altogether, these findings identify and support the importance of CD4+ T cells as a highly potent and clinically important subset for effective T cell-based adoptive therapy. 

### 4.3. Cancer Vaccines

#### 4.3.1. MHC-Class II Epitope Discovery Approaches

The target structures of CD4+ T cells are peptides presented by MHC-class II molecules. Meanwhile, a number of MHC-class II epitopes have been described. As for MHC-class I CD8+ T cell epitopes, they can be derived from tumor-associated antigens (TAAs) or tumor-specific antigens (TSAs). TAAs are non-mutated self-antigens that are overexpressed in tumor cells or expressed in particular organs. TSAs, or neoantigens, can be mutated proteins or unique sequences derived from, e.g., alternative splicing, non-coding regions, or wildtype sequences presented in tumors with deficiencies in the antigen-presentation pathway [109,110,111]. Tumor-specific mutated antigens resemble foreign proteins and therefore seem to be ideal targets, especially for CD4+ T cells [112,113]. Since most of the mutations that accumulate within a tumor cell are unique, such approaches require a high level of individualization, coupled to sophisticated methods like whole genome sequencing and downstream in silico prediction of MHC-binders [109,114].

One limitation in the identification of CD4+ T cell epitopes is that current prediction tools for MHC-class II peptide binding do not perform as precise as for MHC-class I. This is likely due to the variable length of ligands, highly polymorphic allelic products, and degenerated binding motifs for MHC-class II molecules [115,116]. A second problem is that only a small number of mutated peptides will eventually be presented on tumor MHC-molecules, and among them not all will be immunogenic (<1% for MHC-class I, may be more for MHC-class II) [112,117,118]. Finally, individual tumors tend to not express mutations that can be presented on patients’ MHC-allelic products, a phenomenon that is attributed to immunoediting. This is true for MHC-class I [119], but seems to be even more pronounced for MHC-class II [120], pointing out, once again, the importance of CD4+ T cells in the natural antitumor immunity. Hence, tumors with low mutational burden rarely present neoantigens.

Tandem mass spectrometry (MS/MS) of MHC-ligands remains the only method for identifying truly presented tumor peptides. MHC-class II ligands can be isolated from a high number of cancer types [120,121,122]; these ligands do not necessarily originate from the tumor cells themselves and might be also derived from stroma and infiltrating immune cells. Still, among these, differential immunopeptidomics and RNA-seq analyses in benign vs. tumor tissues repeatedly identify tumor-specific sequences [122,123]. The MS/MS approach is especially useful for identifying candidate epitopes, which can be implemented in semi- or fully personalized immunotherapy [114].

#### 4.3.2. Targeting CD4+ T Cells with Vaccines

It is widely accepted that the simultaneous stimulation of both CD8+ and CD4+ T cells is the best strategy for therapeutic vaccination, since, as described above, both cell types are required for a long-lasting immune response and can synergize to fight tumor cells. In early vaccine trials, when candidate tumor-relevant MHC-class II peptides were not yet identified, attempts to provide CD4+ T cell help were carried out by providing foreign proteins like keyhole limpet hemocyanin or tetanus toxoid. Meanwhile, CD4+ T cell activation has evolved as an essential goal of cancer vaccines, whereby two main strategies are used. The first relies on the use of synthetic peptides, either as a mixture of exact MHC-class I and -class II ligands [124,125], or (overlapping) synthetic long peptides (SLPs) [126,127,128,129]. The second approach uses nucleic acids (DNA or RNA) encoding for the relevant antigens or fragments thereof. The advantages and drawbacks of these approaches have been discussed elsewhere [89,130,131].

Although the best anticancer vaccination strategy has not yet been identified (e.g., the vaccine basis, adjuvants or injection scheme), a number of clinical studies have demonstrated that targeting CD4+ T cells is safe. In our experience, antivaccine CD4+ T cell responses are often stronger than CD8+ T cell responses and also last longer, even after vaccination completion [128,132].

However, more importantly, is vaccine-induced CD4+ T cell activation needed, and is it clinically effective?

Cancer vaccine studies are mostly early phase I/II clinical trials that include a limited number of patients and are often non-randomized. It is therefore difficult to specifically address the impact of CD4+ T cells on the clinical outcome. Still, some evidence can be collected in different settings. After treatment of prostate carcinoma patients with the FDA-approved Sipuleucel-T (a whole cell vaccine loaded with the fusion protein prostatic acid phosphatase and GM-CSF), decrease in the PSA serum level was positively correlated with an increased expression of Th1 (and CD8+) genes within tumor infiltrating T cells [133]. More information can be gleaned from vaccines with long peptides, which predominantly activate CD4+ T cells. Pioneer studies using a single (mutated) Ras SLP together with GM-CSF showed robust CD4+ T cell responses and surprisingly long survival of pancreas carcinoma patients after vaccination [134,135]. In a different setting, vaccination with overlapping HPV-16 E6 and E7 peptides emulsified in Montanide ISA-51 was performed in patients with vulvar intraepithelial neoplasia. Both CD8+ and CD4+ antivaccine T cell responses were induced, but complete responders showed significantly higher numbers of HPV-16-specific IFN-γ producing CD4+ T cells than patients with no clinical response [127]. Another very relevant observation of the same group was that a subset of CD4+CD25+FOXP3+ cells, possibly vaccine-specific, was expanded in non-responding patients [136]. This suggests that targeting CD4+ T cells by vaccination might lead to in vivo stimulation of suppressive, instead of effector, CD4+ T cell subsets. We currently do not know if the nature or dose of the antigen, the scheduling of the vaccine applications, and/or patient-specific immune characteristics are impacting the development of antivaccine Tregs; this needs to be more thoroughly investigated. In a randomized melanoma study, MHC-class I and -class II peptides were administered with GM-CSF either alone or together. The CD8+ T cell response rate was clearly lower when patients also received MHC-class II peptides (43% vs. 28%), however, no difference in OS could be observed between study arms [124]. The only parameter that was found to associate with OS was immune response to the MHC-class II peptides, and MHC-class I epitope spreading was detected in a good proportion of these patients [137].

Latest personalized approaches indicate that preferentially CD4+ T cells are induced or boosted in vivo with either RNA- or SLP-based neoantigen vaccines [126,138]. They also suggest that long-term clinical benefit can be reached and that combination with checkpoint Abs could have synergistic effects [125,126,138]. In a larger recent study including 60 selected patients with tumors of high mutational load (NSCLC, melanoma, and bladder carcinoma), a combination of PD-1 blockade with individual multi-SLP vaccines showed an excellent safety profile, and induced the development of CD8+ and CD4+ neoantigen-specific effector T cells (with CD107a cell surface expression in both T cell types) and epitope spreading [139]. Favorable progression free survival times were also noted, but due to the study concept, it could not be attributed to the checkpoint blockade, the vaccine, or the combination of both. All of these recent observations are encouraging, but are not designed for assessing clinical efficacy, and have been performed in different patient cohorts, with different vaccine components, routes, and schedules. Hence, we need to wait for more, when feasible, multiarm trials, to pinpoint the exact role of effector CD4+ T cells in neoantigen-based vaccination.

### 4.4. Immunoediting by CD4+ T Cells?

According to the concept of immunoediting, elimination of tumor cells by the immune system will eventually lead to the emergence of new tumor cell clones able to avoid this attack. Hence, if natural or therapy-induced antitumor CD4+ T cells are meaningful for tumor growth control, tumor cell escape should be detectable. Indeed, as mentioned above, frequent driver mutations were predicted to be generally poorly presented by MHC-II allelic products, and MHC-II allelic products in individual patients have a low predicted binding capacity for peptides derived from mutations expressed in their own tumors [120]. This immune-driven shaping might depend on the sex and age of the patients, since potential MHC-II-mutated ligands were found to be reduced in women and younger patients vs. older men [140]. Following immunotherapy, the loss of CD8+ T cell targets has been documented, however, we do not know yet whether tumor antigens recognized by therapy-induced CD4+ T cells can also become altered or lost. Simultaneous targeting of several antigens should minimize the risk of tumor escape, and ongoing efforts demonstrate that this is feasible in a patient-individualized manner. 

## 5. Conclusions

CD4+ T cells are now recognized as essential and pleiotropic effectors in the antitumor immune response. An increasing number of clinical studies incorporate this knowledge in order to develop more efficient drug products, and evidences are accumulating that CD4+ T cell subsets are the main players in various cancer immunotherapies. However, we still need to learn how to recognize and exploit the many faces of CD4+ T cells. Comprehensive studies of immune cell subsets in selected patient cohorts should increase our understanding of which subpopulations of CD4+ T cells are essential for productive anticancer immune responses, and for response or resistance to immunotherapies. Such advances could not only benefit cancer immunotherapy but also other clinical needs such as pathogen infections or autoimmune diseases. 

## Figures and Tables

**Table 1:** Main CD4+ T cell subsets: markers, cytokines driving their differentiation, and effector cytokines.

**Subset Name**	**Surface Markers**	**Transcription Factors**	**Driving Cytokines**	**Secreted Factors**
**Lineages**				
Th1 [14,15,16]	CXCR3+ CCR6−	T-BET+	IL-12, IFN-γ	IFN-γ, IL-2, TNF
Th2 [13,15,16]	CXCR3− CCR4+ CCR6− CD294+	GATA3+ IRF4+	IL-4	IL-4, IL-5, IL-9, IL-13
Th9 [13,15,16]		IRF4+ PU.1+	IL-4, TGF-β	IL-9
Th17 [14,15,16]	CXCR3− CCR4+ CCR6+ CD161+ IL23R+	IRF4+ ROR-γt+	TGF-β, IL-6, IL-1β, IL-21, IL-23	IL-17, IL-21, IL-22
Th22 [13,14,15,16]	CCR10+ CCR4+ CCR6+	AHR+ FOXO4+	IL-6, TNF, IL-12, IL-23	IL-22
Tregs [13,15,16]	CD127low/− CD25+ CTLA-4+	FOXP3+	TGF-β, IL-2	IL-10, TGF-β
CD4+ CTLs [17]	CD107a/b+ NKG2A+ NKG2D+ CRTAM+ CD57+			Granzyme B, perforin
GC-Tfh [15,18]	CXCR5+ ICOS+ PD-1+	BCL6+	IL-12, IL-23, TGF-β	IL-21, IL-4
cTfh [15,18]	CXCR5+ ICOS+ PD-1+	BCL6−		Il-21, IL-10, IL-2
**Differentiation stages**				
T_N_ [15,16,19]	CD45RA+ CD45RO− CCR7+ CD95− CD27+ CD62L+			
T_SCM_ [15,16,19]	CD45RA+ CD45RO− CCR7+ CXCR3+ CD95+ CD27+ CD62L+			
T_CM_ [13,15,16]	CD45RA− CD45RO+ CD95+ CCR7+ CD27+ CD62L+			
T_EM_/T_RM_ [13,15,16]	CD45RA− CCR7− CD27low CD62Llow/−			
T_EMRA_ [20]	CD45RA+ CCR7− CD62Llow/−			

Subset abbreviations: Th = T helper; Treg = regulatory CD4+ T cell, CTL = cytotoxic T lymphocyte, GC-Tfh = germinal center T follicular helper cell, cTfh = circulating T follicular helper cell, T_N_ = naïve T cell, T_CM_ = central memory T cell, T_SCM_ = T memory stem cell, T_EM_ = T effector memory cell, T_RM_ = tissue-resident memory T cell, T_EMRA_ = T effector memory cell re-expressing CD45RA. Abbreviations for markers, transcription factors and cytokines: AHR = aryl hydrocarbon receptor, BCL6 = B-cell lymphoma 6, CRTAM = cytotoxic and regulatory T cell molecule, CTLA-4 = cytotoxic T-lymphocyte-associated protein 4, C(X)CR = chemokine receptor, FOXP3 = forkhead box p3, FOXO4 = Forkhead box protein O4; GATA3 = GATA binding protein 3, ICOS = inducible T-cell costimulator, IL = interleukin, IFN = interferon, IRF4 = interferon regulatory factor 4, NKG2A/D = natural killer group 2 member A/D, PD-1 = programmed cell death protein-1; PU.1 = purine-rich box-1, ROR-γt = RAR-related orphan receptor gamma, T-BET = T-box protein expressed in T cells, TGF-β = transforming growth factor β, TNF = tumor necrosis factor. References are given in uppercase brackets.
